# Type 2 diabetes mellitus negatively affects the functional performance of 6-min step test in chronic heart failure: a 3-year follow-up study

**DOI:** 10.1186/s13098-024-01464-z

**Published:** 2024-09-14

**Authors:** Aldair Darlan Santos-de-Araújo, Daniela Bassi-Dibai, Izadora Moraes Dourado, Cássia da Luz Goulart, Renan Shida Marinho, Jaqueline de Almeida Mantovani, Gabriela Silva de Souza, Polliana Batista dos Santos, Meliza Goi Roscani, Shane A. Phillips, Audrey Borghi-Silva

**Affiliations:** 1 Cardiopulmonary Physiotherapy Laboratory, Universidade Federal de São Carlos, Federal University of Sao Carlos Rodovia Washington Luiz, São Carlos, SP 13565-905 Brazil; 2 Management in Health Programs and Services, Universidade CEUMA, São Luís, MA Brazil; 3 Health Sciences and Technologies, Universidade de Brasília, Brasília, DF Brazil; 4 Inter-Units of Bioengineering, University of São Paulo, São Carlos, SP Brazil; 5 Morgana Potrich Faculty, Mineiros, GO Brazil; 6 Department of Medicine, Universidade Federal de São Carlos (UFSCar), Sao Carlos, SP Brazil; 7 Department of Physical Therapy, College of Applied Health Sciences, University of Illinois Chicago, Chicago, IL USA

**Keywords:** Step test, Prognosis, Chronic heart disease, Diabetes, Mortality

## Abstract

**Background:**

Type 2 diabetes mellitus (T2DM) and chronic heart failure (CHF) present a decrease in functional capacity due to the intrinsic nature of both pathologies. It is not known about the potential impact of T2DM on functional capacity when assessed by 6-min step test (6MST) and its effect as a prognostic marker for fatal and non-fatal events in patients with CHF.

**Objective:**

to evaluate the coexistence of T2DM and CHF in functional capacity through 6MST when compared to CHF non-T2DM, as well as to investigate the different cardiovascular responses to 6MST and the risk of mortality, decompensation of CHF and acute myocardial infarction (AMI) over 36 months.

**Methods:**

This is a prospective cohort study with 36 months of follow-up in individuals with T2DM and CHF. All participants completed a clinical assessment, followed by pulmonary function testing, echocardiography, and 6MST. The 6MST was performed on a 20 cm high step and cardiovascular responses were collected: heart rate, systemic blood pressure, oxygen saturation, BORG dyspnea and fatigue. The risk of mortality, acute myocardial infarction and decompensation of CHF was evaluated.

**Results:**

Eighty-six participants were included. The CHF-T2DM group had a significantly lower functional capacity than the CHF non-T2DM group (p < 0.05). Forced Expiratory Volume in one second (L), ejection fraction (%), gender and T2DM influence and are predictors of functional capacity (p < 0.05; adjusted R squared: 0.419). CHF-T2DM group presented a higher risk of mortality and acute myocardial infarction over the 36 months of follow-up (p < 0.05), but not to the risk of decompensation (p > 0.05).

**Conclusion:**

T2DM negatively affects the functional performance of 6MST in patients with CHF. Gender, ejection fraction (%), FEV1 (L) and T2DM itself negatively influence exercise performance.

## Introduction

Common, highly prevalent, closely related and frequently associated, CHF and T2DM have a bidirectional relationship, that is, the origin and evolution of each pathology can be mutually influenced [1–4]. The interactions between both diseases is widely known [5, 6], however, the treatment approach continues to be a challenge, from screening to optimizing therapeutic decision-making that addresses aspects of rehabilitation of these individuals due to the high rate of morbidity and mortality and the heterogeneous clinical presentation of both conditions [1, 6, 7].

In both diseases, exercise capacity has been adopted as an important outcome and the main guidelines and international campaigns have drawn attention to the importance of this assessment and the inclusion of this outcome in therapeutic optimization [8–11]. Since the results that reflect exercise capacity have discriminative prognostic value for mortality risk, risk of unfavorable outcomes, the assessment of treatment efficacy in both conditions is highly desirable [12–14].

When it comes to exercise capacity, cardiopulmonary exercise testing (CPET) has established itself as the most effective tool for assessing this outcome [10, 15], however, the arsenal of equipment used and the need for a team that involves trained professionals led scientists to develop options that could reflect the CPET's ability to exercise in a more economically accessible and simple, but not replaceable [16, 17]. The 6-min step test (6MST) has gained notoriety and clinical and scientific popularity due to its practicality and simple, low-cost and easily available alternative option, especially in environments where the most sophisticated resources and equipment for achieving the gold standard are not available, in addition to having an important correlation with CPET [16, 18]. In both individuals with CHF [16] and individuals with T2DM [18], the reliability and validity of the 6MST has already been scientifically proven and the test has strong concurrent validity when compared to the CPET.

Undoubtedly, both disease negatively impact on exercise capacity, affecting the cardiovascular, respiratory and metabolic dynamic for the supply of oxygen to peripheral muscles [19, 20]. However, although the coexistence of T2DM and CHF has been growingly reported, description of fatal and nonfatal events and its relation with functional (in)capacity considering the presence of both conditions is still scarce. Therefore, the objective of this investigation is to evaluate whether individuals with CHF with T2DM have worse functional capacity when compared to a group with CHF without T2DM. Secondarily, we aimed to investigate the different cardiovascular responses presented in both groups and the risk of mortality, decompensation of heart failure and acute myocardial infarction over 36 months. Our hypothesis is, based on all the previously described aspects, that T2DM not only negatively affects the functional performance of individuals with CHF, but also presents itself as an independent factor for reducing functional capacity in the 6MST, worse cardiovascular responses and presents greater mortality, decompensation of CHF and acute myocardial infarction risk.

## Methodology

### Study design

This is a prospective longitudinal investigation with a follow-up of 3 years (36 months) carried out by the Cardiopulmonary Physiotherapy Laboratory (LACAP) of the Federal University of São Carlos—UFSCar, located in São Carlos, SP, Brazil. The participant recruitment process took place between December 2017 and November 2020. The university's ethics committee previously approved the development of the investigation under protocol number 5.188.654 and the research followed the principles of the Declaration of Helsinki. The STrengthening the Reporting of OBservational Studies in Epidemiology (STROBE) guideline was used to conduct the study [21]. All participants were informed about the research objectives and gave their informed consent before being evaluated***.***

### Participants

The Cardiology Outpatient Clinics of the Medical Specialties Center (CEME) and the São Carlos University Hospital (HU-UFSCar) were used to actively search for participants eligible for the investigation. We included patients over 40 years of age, confirmed diagnosis of heart failure with left ventricular ejection fraction below 50% by echocardiography, with or without clinical diagnosis of T2DM, clinical stability and absence of medication changes in the last 3 months. Those aged over 80 years, diagnosed with heart failure with preserved ejection fraction, history of cardiovascular events in the last 6 months, decompensation of the disease in the last 3 months, presence of any implantable cardiac pacemaker, unstable angina, diagnosis of any neoplasms, uncontrolled systemic arterial hypertension, cognitive impairment or lack of understanding of the study proposal were excluded of study.

### Initial assessment

Initially, a prior anamnesis was carried out using an assessment form developed by the laboratory and researchers involved so that personal information, associated pathologies and medications used were collected. The medical records of the included patients were also used as a tool to search for important information.

### Anthropometric variables

To estimate the height of participants, a stadiometer (Welmy R-110, Santa Bárbara do Oeste, São Paulo, Brazil) was used. Body mass in kilograms (kg), body fat mass (kg), body fat percentage (%) and skeletal muscle mass (kg) were determined through bioelectrical impedance analysis, using the InBody 720 device. Participants were instructed to fast for at least 4 h, wear light clothing, remove all metallic objects in contact with the body, urinate before the exam, avoid drinking alcoholic beverages for 12 h and not perform strenuous physical exercise the day before the evaluation. During the examination, participants were positioned in an upright position, barefoot, with their shoulders slightly abducted and their elbows flexed at approximately 15°, as recommended by the manufacturer (BIOSPACE, 2004). The Body Mass Index (BMI) was calculated by dividing body mass (kg) by height squared in meters (kg/m^2^). The BMI classification was established as follows: low weight (15–19.9 kg/m^2^); normal weight (20–24.9 kg/m^2^); overweight (25–29.9 kg/m^2^); obesity I (30–34.9 kg/m^2^); obesity II (35–39.9 kg/m^2^); and obesity III (≥ 40 kg/m^2^)[22].

### Minnesota Questionnaire

Previously validated for the Brazilian population [23], this questionnaire consists of 21 questions relating to the limitations associated with heart failure considering the last month. The answers to each question range from 0 to 5, where 0 represents no limitations and 5 the maximum limitation. These questions involve a physical dimension (1–7, 12 and 13), which are highly related to dyspnea, fatigue; emotional dimension (17- 21); and other issues (number 8, 9, 10, 11, 14, 15 and 16) which, together with the previous dimensions, form the total score.

### New York Heart Association—NYHA

The New York Heart Association (NYHA) functional classification was used to assess the severity of functional limitations resulting from the CHF condition based on the symptoms experienced by the participant during physical activity. It allows stratifying the degree of limitation imposed by it: class I—absence of symptoms during daily activities, with limitation in efforts similar to that expected in healthy individuals; class II—symptoms triggered by daily activities; class III—symptoms triggered by activities less intense than everyday activities; class IV—symptoms at rest [24].

### Pulmonary function—spirometry

The assessment of lung function was conducted using spirometry (Masterscreen Body, Mijnhardt/Jäger, Würzburg, Germany) by a previously trained researcher, following conventional techniques and the acceptability and reproducibility guidelines of the American Thoracic and European Respiratory Societies (ATS/ERS). At least three slow and forced maneuvers considered acceptable and reproducible were performed, as recommended, and repeated 20 min after administration of 400 µg of Albuterol Sulfate. Participants with overlapping chronic obstructive pulmonary disease were diagnosed according to the GOLD criteria (post-bronchodilator forced expiratory volume in one second (FEV1)/forced vital capacity (FVC) ratio < 0.70) [25, 26].

### Transthoracic echocardiogram

The transthoracic echocardiogram was performed by a cardiologist, using an ultrasound device with a 3 MHz transducer (Phillips, HD11 XE, Bothell, Washington, United States) according to recommendations [27]. The end systolic and diastolic diameter of the left ventricle, early diastolic mitral filling velocities (E wave), early diastolic velocity of the mitral annulus (E' wave) and left ventricular ejection fraction (LFEV) were obtained using the Simpson method [28].

### 6-min step test—6MST

The 6MST has been previously validated for individuals with CHF [16]. Prior to the test, upon arrival at the laboratory, participants were informed about the nature and dynamics of the test so that any doubts regarding carrying it out could be clarified. Then, they underwent a period of 4 min of rest (2 min sitting and 2 min standing) so that vital signs could be collected (resting heart rate [HR], peripheral oxygen saturation [SpO_2_] and blood pressure systemic) in addition to perceived exertion for dyspnea and fatigue of the lower limbs using the BORG 10 scale in each position. At the end of the 4 min, they were instructed to go up and down a single step with a height of 20 cm (cm) in a self-paced manner, being allowed to slow down, if necessary, and even interrupt the test to rest. Verbal encouragement commands were used for each minute of testing and the time remaining until completion. The step numbers were counted from the beginning to the end of the 6-min time and recorded. Vital signs collected prior to the test, as well as feelings of lower limb fatigue and dyspnea were obtained immediately at the end of the test and the 6th min of recovery.

Despite being considered a test of a submaximal nature, some criteria for interrupting the exam were adopted so that the integrity of the patient's health was guaranteed: reaching 85% of maximum HR, arterial oxygen saturation ≤ 87%, systolic blood pressure (SBP) greater than 170 mmHg and DBP greater than 110 mmHg, BORG score greater than 7 for dyspnea and lower limb fatigue, anginal pain > 2, dizziness, vertigo and nausea. The prediction of functional performance of participants in the 6MST for the Brazilian population was made using the equations proposed by Arcuri et al.[29] 6MST = 209 – (1.05 × age) for men and 6MST = 174 – (1.05 × age) for women, where age is expressed in years; and Albuquerque et al.[30] 6MST = 106 + (17.02 × [0:woman; 1:man]) + (− 1.24 × age) + (0.8 × height) + (− 0.39 × weight) where 6MST is expressed in number of steps; age, in years; height, in cm; and weight, in kg.

### Participants follow-up

Information on mortality, AMI and acute decompensated heart failure was collected through periodic telephone calls every 6 months and/or through hospital records from the date of the patient's initial evaluation in the laboratory. According to the European Society of Cardiology [28], acute decompensation of heart failure was understood as a rapid or gradual clinical presentation of the signs and symptoms of heart failure at rest, severe enough to cause unplanned office visits, emergency room visits or hospitalization requiring urgent assessment and subsequent initiation or intensification of treatment that includes therapies or procedures.

### Statistical analyses

Data are presented as mean and standard deviation or absolute values and percentages of occurrence when appropriate. The Kolmogorov–Smirnov test was used to verify the normality of the data. For the analysis between the groups test T for independent samples was used when the data presented a normal distribution. When the data presented a non-parametric distribution, the Mann–Whitney test was used. The χ^2^ test was used to compare categorical variables. Kaplan–Meier analysis was used to test the risk of all-cause mortality, acute decompensated heart failure, and acute myocardial infarction over 36 months of follow-up. Differences between curves were evaluated using the Log-rank test, Breslow and Tarone-Ware.

The covariates included in the present analysis constitute a broad spectrum of factors associated with unfavorable outcomes (mortality, decompensation of heart failure and AMI). Univariate linear regression analyses were performed to verify the association between the independent variables and the dependent variable (steps in the 6MST) [31]. For the multiple linear regression model, variables that presented a p-value < 0.20 in the univariate analysis were selected as covariates [32]. Comparisons of 6MST performance and cardiovascular responses between groups were expressed as mean, standard deviation (SD), mean difference (MD), and effect size calculated using Cohen’s d, with the categorization based on the values established by Cohen [33]. The effect size was calculated based on the Cohen d, according to the website: <https://www.psychometrica.de/effect_size.html>. It was considered the following interpretation of the d value: 0.2 (weak), 0.5 (moderate) and > 0.8 (large effect size) [33]. Raincloud plots were produced using the JASP 0.18.2 software [34] for data visualization of the step test performance and predictive values <https://jasp-stats.org/>. All analyzes were performed using GraphPad Software, Inc. (2019). *GraphPad Prism* (versão 8.0.1). San Diego, CA <https://www.graphpad.com>. The probability of type 1 error occurrence was established at 5% for all tests (p < 0.05).

## Results

**Fig. 1:**
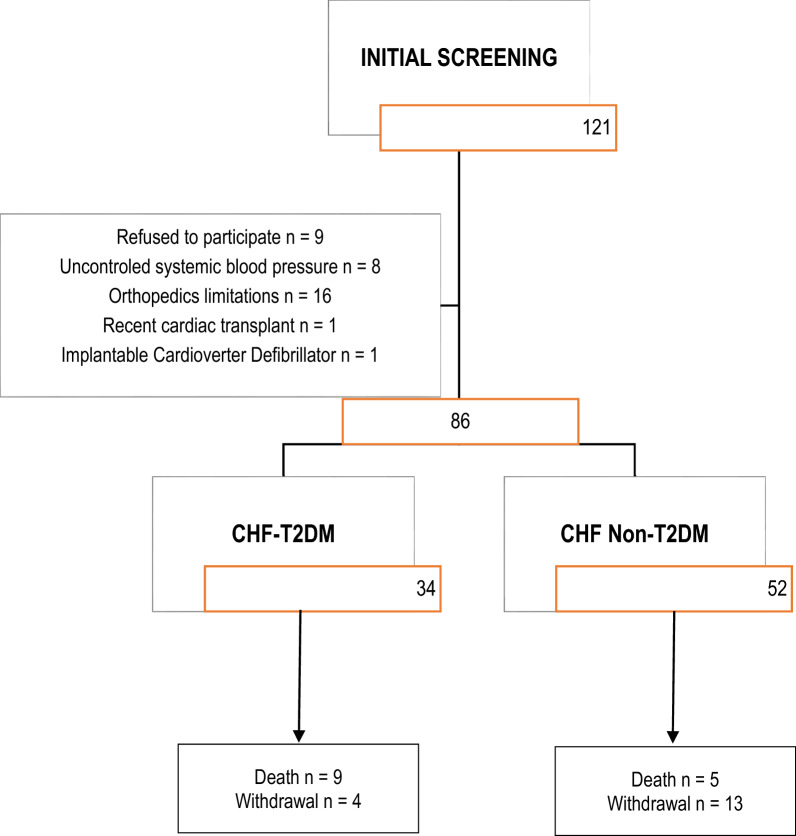
Flowchart

Initially, one hundred and twenty-one participants were recruited, however thirty-five were not included. Finally, eighty-six participants were included: 34 CHF-T2DM group and 52 in CHF non-T2DM group (Fig. 1). Information about the characteristics of the sample included in the study can be viewed in Table 1. The groups did not differ in terms of age (years), sex distribution and height (m). However, the CHF-T2DM group had higher body weight and BMI when compared to the CHF non-T2DM and, consequently, a greater number of participants with obesity (65%) (class I [40%], class II [18%] and class III [5%]). Additionally, this same group had a higher prevalence of coronary artery disease (12%), dyslipidemia (72%), use of beta-blockers (85%) and lower LVEF (%). No statistically significant differences were observed in the outcomes of quality of life (Minnesotta questionnaire), functional classification (NYHA) and lung function (spirometry). In total, 14 individuals died over the 36 months of follow-up (9 in the CHF-T2DM group and 5 in the CHF non-T2DM). Furthermore, 13 individuals progressed to acute decompensation of heart failure and 10 to AMI.

**Table 1:** General characteristics of the study sample at initial assessment (n = 86)

Variables	All volunteers (n = 86)	CHF Non-diabetics (n = 52)	CHF Diabetics (n = 34)	P value
Age (years)	62 ± 11	62 ± 12	62 ± 11	0.688
Gender				
Male, n (%)	64 (74)	41 (79)	23 (68)	0.181
Female, n (%)	22 (26)	11 (21)	11 (32)	
Body Mass (kg)	78.82 ± 17.89	75.27 ± 16.49	84.24 ± 18.80	0.022*
Height (m)	1.66 ± 0.09	1.66 ± 0.08	1.65 ± 0.10	0.709
BMI (kg/m^2^)	28.72 ± 6.07	27.36 ± 5.72	30.79 ± 6.09	0.010*
Normal	24 (28)	18 (35)	6 (18)	0.009*
Overweight	27 (31)	21 (40)	6 (18)	
Obesity class I	22 (26)	8 (15)	14 (41)	
Obesity class II	10 (12)	4 (8)	6 (18)	
Obesity class III	3 (3)	1 (2)	2 (5)	
Death patients, n (%)	14 (16)	5 (10)	9 (26)	0.038*
COVID-19, n (%)	2 (14)	1 (20)	1 (11)	
Decompensation, n (%)	8 (58)	4 (80)	4 (45)	
Renal insufficiency, n (%)	1 (7)	0 (0)	1 (11)	
Diabetes complication, n (%)	1 (7)	0 (0)	1 (11)	
AMI, n (%)	2 (14)	0 (0)	2 (22)	
Decompensation, n (%)	13 (15)	5 (10)	8 (24)	0.078
AMI, n (%)	10 (12)	3 (6)	7 (21)	0.036*
Risk factors, n (%)				
Atrial Fibrillation	18 (20)	8 (15)	10 (29)	0.117
Asma	11 (13)	4 (8)	7 (21)	0.061
Atherosclerosis	3 (3)	1 (2)	2 (6)	0.344
Coronary artery disease	5 (6)	1 (2)	4 (12)	0.047*
Hypertension	65 (76)	42 (81)	23 (67)	0.344
Depression	16 (19)	9 (17)	7 (21)	0.605
COPD	21 (24)	13 (25)	8 (24)	0.877
Obesity	35 (41)	13 (25)	22 (65)	< 0.001*
Dyslipidemia	40 (47)	17 (33)	23 (72)	< 0.001*
Deep vein thrombosis	4 (5)	1 (2)	3 (9)	0.119
Stress	20 (23)	15 (29)	5 (15)	0.167
Type 2 Diabetes				
Alcoholism	5 (6)	4 (8)	1 (3)	0.390
Thyroid Disease	12 (14)	7 (13)	5 (15)	0.783
Obstructive Sleep Apnea Syndrome	9 (10)	7 (13)	2 (6)	0.299
Current Smokers	14 (16)	10 (19)	4 (12)	0.421
Ex-smokers	42 (49)	25 (48)	17 (47)	0.653
Minnesota questionnaire		27.45 ± 21.34	31.66 ± 19.73	0.378
NYHA, n (%)				
I	35 (41)	20 (38)	15 (44)	0.603
II	35 (41)	24 (46)	11 (32)	
III	14 (17)	7 (13)	7 (21)	
IV	2 (2)	1 (2)	1 (3)	
Pulmonary Function				
FEV1 (L)	2.40 ± 0.74	2.45 ± 0.77	2.32 ± 0.69	0.473
FEV1 (%)	76.00 ± 30.73	75.28 ± 32.98	77.13 ± 27.26	0.789
FVC (L)	3.30 ± 0.89	3.40 ± 0.95	3.15 ± 0.81	0.216
FVC (%)	91.12 ± 17.59	92.71 ± 18.88	88.75 ± 15.40	0.335
FEV1/FVC	0.66 ± 0.24	0.64 ± 0.26	0.69 ± 0.21	0.343
Echocardiogram				
LV end-diastolic diameter (mm)	58.15 ± 11.00	57.38 ± 10.46	59.44 ± 11.94	0.444
LV end-systolic diameter (mm)	46.52 ± 11.66	44.98 ± 11.07	49.08 ± 12.38	0.159
Mitral E wave (cm/s)	72.46 ± 24.20	69.86 ± 23.26	77.67 ± 25.89	0.268
Mitral E’ wave (cm/s)	7.43 ± 2.49	7.93 ± 2.36	6.53 ± 2.53	0.063
LVEF, %	39.60 ± 8.20	41.29 ± 7.81	37.03 ± 8.23	0.018*
Medications, n (%)				
SABA	7 (8)	4 (8)	3 (9)	0.786
LABA	5 (6)	2 (4)	3 (9)	0.298
LAMA	2 (2)	2 (4)	0 (0)	0.262
Bronchodilator	12 (14)	8 (15)	4 (12)	0.714
ACE inhibitors	55 (64)	31 (60)	24 (70)	0.300
Calcium channel blocker	1 (1)	0 (0)	1 (3)	0.728
Diuretics	66 (77)	37 (71)	29 (85)	0.129
Oral hypoglycemic agents	26 (30)	0 (0)	26 (76)	< 0.001*
Anticoagulant	53 (62)	33 (63)	20 (62)	0.923
Digoxin	14 (17)	9 (17)	5 (15)	0.890
Beta blocker	69 (81)	40 (77)	29 (85)	0.049*
Statins	35 (41)	18 (35)	17 (48)	0.071

Values are mean ± SD or absolute values (%)

*CHF* chronic heart failure, %: percentage, *kg* kilos, *m* meter, *BMI* body mass index, acute myocardial infarction, *COPD* Chronic Obstructive Pulmonary Disease, *DASI* Duke Activity Status Index, *VO2* oxygen uptake, *NYHA* New York Heart Association, *FEV*_*1*_ forced expiratory volume in 1 s, *L* liters, *FVC* forced vital capacity, *LV* left ventricular; millimeter, *cm* centimeter, *Mitral E/E’ ratio* early diastolic mitral filling velocity/ early diastolic mitral annular velocity, *LVEF* left ventricular ejection fraction, *SABA* short-acting β-agonist, *LABA* long-acting β-agonist, *LAMA* long-acting muscarinic antagonists, *ACE* angiotensin-converting inhibitors

*p < 0.05 Statistical significance for Student’s t-test, Mann–Whitney test or χ^2^ test

**Fig. 2:**
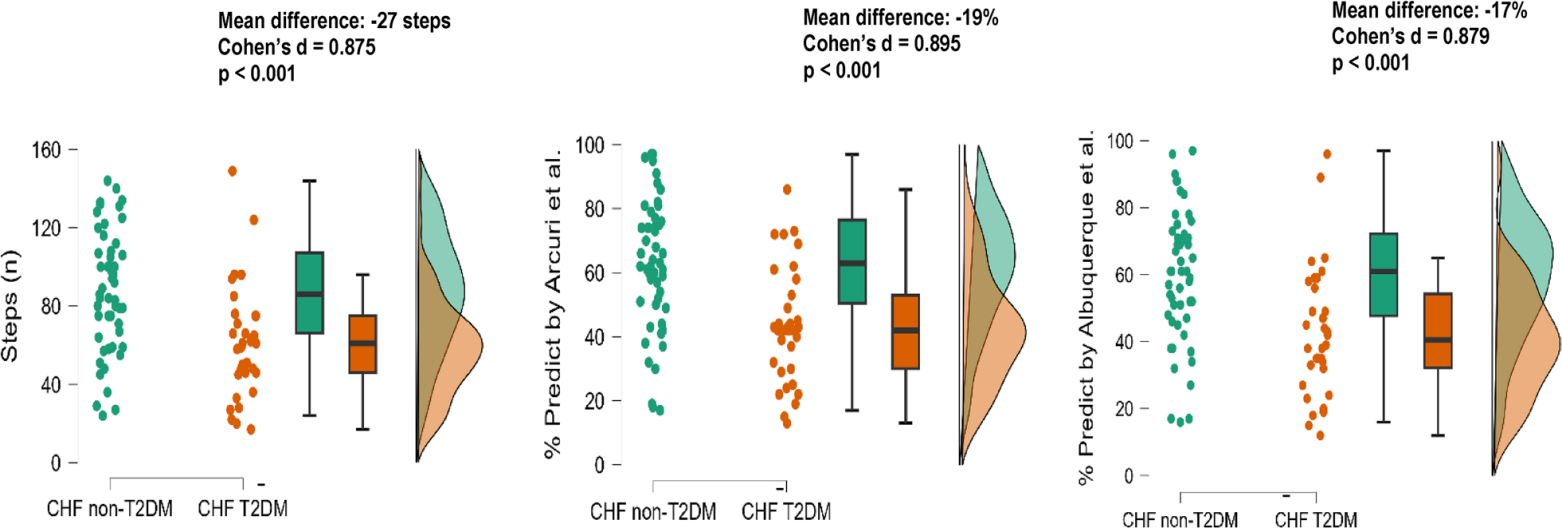
Raincloud plots for functional capacity by 6MST and predicted values in CHF non-T2DM and CHF-T2DM. *CHF non-T2DM* chronic heart failure without type 2 diabetes mellitus, *CHF-T2DM* chronic heart failure with type 2 diabetes mellitus, % percentage, *n* number in absolute value, p < 0.05: statistical significance

In Fig. 2, when we evaluated functional performance comparing to 6MST in both groups, we observed that the CHF-T2DM group had a significantly lower functional capacity than the CHF non-T2DM group (60 ± 29 versus 87 ± 31; Cohen’s d = 0.875) and that they achieved an average percentage of 45 ± 20 versus 64 ± 22 when considering the prediction equation by Arcuri et al., and 43 ± 19 versus 60 ± 19 when considering the prediction equation by Albuquerque et al. Regarding cardiovascular responses (Table 2), we only found a lower heart rate chronotropic response by heart rate in beats per minute in the CHF-T2DM (bpm) at peak exercise (97 ± 26 versus 108 ± 21; Cohen’s d: 0.476).

**Table 2:** Cardiovascular responses through 6MST

Variables	CHF Non-T2DM n = 52)	CHF T2DM (n = 34)	Mean Difference	Cohen D	P value
HR (bpm) rest	72 ± 12	68 ± 12	− 4.00	0.333	0.144
HR (bpm) peak	**108 ± 21**	**97 ± 26**	**− 11.00**	**0.476**	0.030*
HR (bpm) rec 1’	− 20 ± 17	− 17 ± 17	− 3.00	0.176	0.392
SBP (mmHg) rest	118 ± 12	121 ± 16	3.00	0.219	0.367
SBP (mmHg) peak	147 ± 20	146 ± 25	− 1.00	0.045	0.939
DBP (mmHg) rest	78 ± 9	79 ± 9	− 1.00	0.111	0.859
DBP (mmHg) peak	86 ± 19	89 ± 15	− 3.00	0.171	0.497
SpO_2_ (%) rest	96 ± 2	96 ± 2	0.00	0.000	0.907
SpO_2_ (%) peak	95 ± 3	95 ± 3	0.00	0.000	0.821
BORG Dyspnea rest	0.1 ± 1.00	0.5 ± 1.00	− 0.40	0.238	0.112
BORG Dyspnea peak	3 ± 2	3 ± 3	0.02	0.00	0.989
BORG fatigue lower limbs rest	0.1 ± 0.7	0.1 ± 0.5	− 0.02	0.00	0.837
BORG fatigue lower limbs peak	3 ± 3	3 ± 2	− 0.30	0.00	0.589

Values are mean ± Standard Deviation

*CHF* chronic heart failure, *6MST* six-minute step test, % percentage, *HR* heart rate, *bpm* beats per minute, *rec* recovery, *SBP* systolic blood pressure, *mmHg* millimeters of mercury, *DBP* diastolic blood pressure, *SpO2* peripheral oxygen saturation

*Statistical difference between groups highlighted in bold (p < 0.05)

**Fig. 3:**
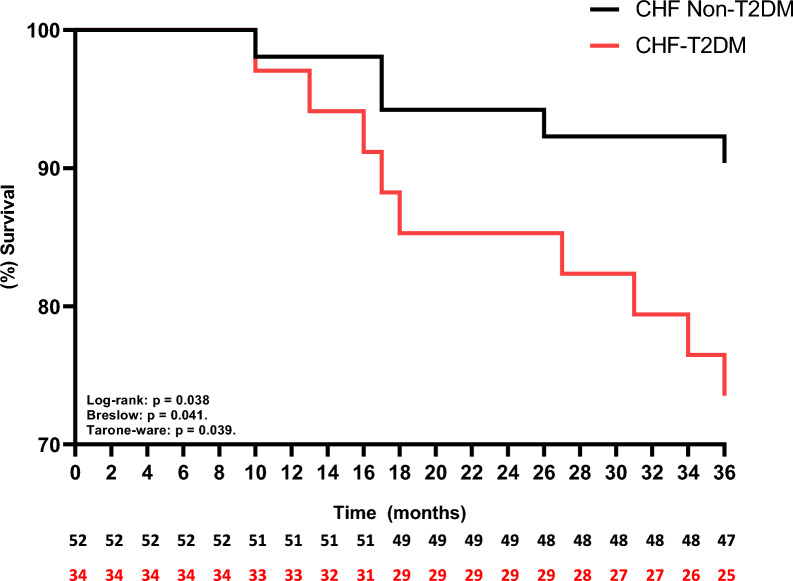
Kaplan–Meier curve for mortality over a period of 36 months. *CHF* chronic heart failure, %: percentage

**Fig. 4:**
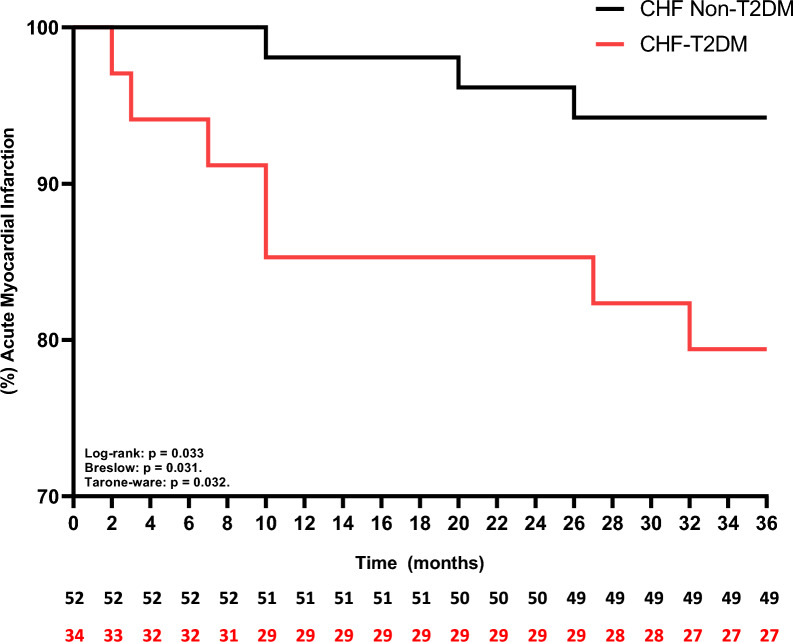
Kaplan–Meier curve for acute myocardial infarction over a period of 36 months. *CHF* chronic heart failure, % percentage

**Fig. 5:**
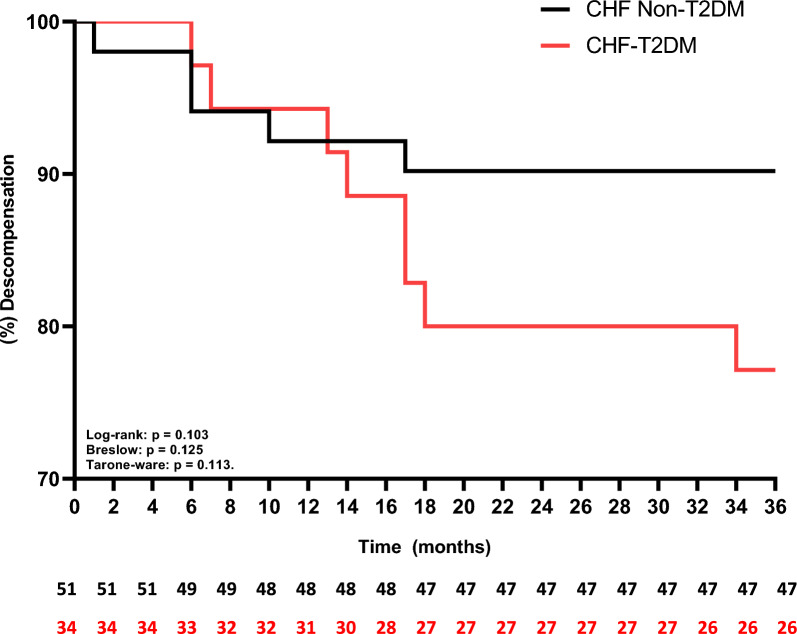
Kaplan–Meier curve for acute decompensation over a period of 36 months. *CHF* chronic heart failure, % percentage

The univariate linear regression model (Table 3) revealed that FEV1 (L), ejection fraction (%), gender and T2DM influence and are predictors of approximately 42% functional capacity (p < 0.05; adjusted R squared: 0.419). Secondarily, when we analyzed the Kaplan–Meier curves, we observed that the CHF-T2DM presented a higher risk of mortality (Fig. 3) and acute myocardial infarction (Fig. 4) over the 36 months of follow-up (p < 0.05 to Log-rank, Brelow and Tarone-ware), however, regarding the risk of heart failure decompensation (Fig. 5), there was no statistically significant difference between the groups (p > 0.05 to Log-rank, Brelow and Tarone-ware).

**Table 3:** Stepwise multiple linear regression model for the six-minute step test

Dependent variable: steps from 6MST						
Variables	β	Std. error	t	p value	CI 95%	
Intercept	− 36.258	18.952	− 1.913	0.060	− 74.039, 1.523	
FEV1 (L)	25.583	4.381	5.840	0.008	16.851, 34.316	
Ejection Fracion (%)	0.875	0.411	2.125	0.037	0.054, 1.695	
[T2DM = 0]	18.990	6.152	3.087	0.003	5.556, 36.191	
[T2DM = 1]	0^a^					
[Gender = 0 Female]	20.873	4.381	5.840	< 0.001	16.851, 34.316	
[Geeder = 1 Male]	0^a^					

R squared 0.450; Adjusted R Squared: 0.419

*6MST* six-minute step test, *FEV* forced expiratory volume, *L* liter, *m* meter, % percentage, *T2DM* diabetes mellitus type 2, β beta, *std* standard, *CI* confidence interval

^a^This parameter is set to zero because it is redundant

## Discussion

The main results of this investigation are associated with some important aspects: (1) for the first time, the impact of T2DM on CHF was investigated considering the performance and cardiovascular variables of 6MST; (2) we confirmed our hypothesis that the association of T2DM and CHF presents worse functional capacity compared to the CHF non-T2DM group; (3) secondarily, we observed a higher risk of mortality and AMI in the CHF-T2DM over 36 months of follow-up.

The heterogeneous presentation of CHF, that is, concomitant with other risk factors that contribute to the increase in unfavorable outcomes, with consequent development of disabling functional limitations [28, 35]. Particularly, in individuals affected by CHF, the decrease in functional capacity is linked to multifactorial mechanisms that involve, above all, early anaerobic metabolism resulting from a combination of reduced blood flow in skeletal muscle, decreased aerobic enzymes in skeletal muscle, morphological and functional changes of musculoskeletal fibers and inefficiency of the cardiovascular and respiratory system [36–38]. T2DM, in turn, presents peculiar characteristics that compromise exercise capacity in this population, mainly associated with ineffective glucose uptake, mitochondrial imbalance and the transition from oxidative to glycolytic fiber type [39, 40].

Paradoxically to the physiological limitations mentioned above, the effort required to perform the 6MST requires vertical displacement and the involvement of large muscle groups that demand greater cardiovascular stress when compared, for example, to the 6-min walk test, leading to an increase extraction oxygen [16, 29]. Considering oxygen uptake, it is nothing new that, individually, both diseases present a decrease in functional capacity when evaluated by field and laboratory tests. In individuals with CHF, whether with a reduced ejection fraction or with its preservation, different methods that reflect this outcome indicate functional impairment over time [41, 42]. The same reasoning can be observed in patients with T2DM [40, 43].

Previously, and in an unprecedented way, an investigation proved that in individuals with CHF the addition of T2DM is associated with a reduction in the distance covered during the 6-min walk test (6MWT) in addition to being an independent determinant of worse performance in the group with coexistence of both pathologies [44]. Still considering the phenotypic nature of CHF presentation, recently, a group of researchers observed that T2DM demonstrated to be the strongest predictor of limited exercise capacity in CHF and preserved ejection fraction when also assessed by the 6MWT [45].

In healthy individuals, variables such as weight (kg), height (cm), age (years) and gender influence 6MST performance and explained at least 42% of the variability in functional capacity [30]. Parallel to this, our results point to an influence of gender, T2DM, ejection fraction (%) and FEV1 (L) and, undoubtedly, we need to recognize how much each variable makes sense in our regression model since current literature has demonstrated the influence of each of them on exercise capacity. The influence of gender is associated with the nature of the physiological difference that men and women present in the cardiovascular, respiratory and musculoskeletal systems, both in healthy individuals and in individuals affected by heart failure [46, 47]. In turn, airflow limitation, more specifically when assessed by FEV1 (L), contributes to functional performance in this population also being compromised. The contribution of lung function to exercise capacity in patients with CHF has been previously discussed and accounts for approximately 30% of maximal exercise capacity during CPET [48].

Our sample presented some important characteristics that deserve discussion. At first, we must keep in mind that the majority of people affected by T2DM are overweight or obese [3]. Although there is a paradoxical relationship, that is, inversely proportional, between weight gain and 6MST performance, in practical terms, when we talk about T2DM it is practically utopian to disregard overweight or obesity in this population due to the close relationship between these two outcomes [30]. We minimally understand the importance of controlling the variable that reflects obesity in both groups so that this bias is minimized, but this may reflect a small portion of the population affected by CHF-T2DM since the coexistence of both pathologies is highly prevalent [49] and that obesity is strongly connected to T2DM [3]. Nevertheless, the impact of T2DM culminates in structural and functional changes in the heart muscle that lead to exercise intolerance in patients with CHF and, not surprisingly, the CHF-T2DM group showed a lower left ventricular ejection fraction that may be a reflection of coexistence of both diseases [20].

When we considered the characterization of our sample using the NYHA scale, we observed that most participants were categorized as NYHA I and II. Curiously, there was a significant risk of mortality and AMI in our sample, revealing a true paradox, as these functional classes typically reflect better functional capacity. Since our patients were followed during a pandemic period, we hypothesize several possible explanations for these unfavorable outcomes: (a) COVID-19 infection and its deleterious effects, leading to an increased risk of AMI and mortality [50]; (b) the impact of lockdown on increasing the risks associated with these outcomes [51]; (c) limited discrimination of the NYHA classification [52].

Clinically, our results contribute not only to recognizing the impact of T2DM in individuals with CHF on 6MST performance, but mainly so that the results can be used in more precise therapies that consider the nature of the coexistence of both pathologies once the evaluation of this outcome. It is routinely used for prognostic, diagnostic, pharmacological optimization, monitoring of disease progression and investigation of functional decline.

## Limitations

This is a study with some limitations that deserve to be described. It was not possible to characterize the sample according to metabolic outcomes such as fasting blood glucose or glycated hemoglobin and we also do not have information about the time of diagnosis of T2DM.

## Conclusion

T2DM negatively affects the functional performance of 6MST in patients with CHF. Sex, ejection fraction (%), FEV1 (L) and T2DM itself negatively influence this outcome and must be considered within the evaluation.

## Data Availability

The set of data generated and/or analyzed during the present study are available through the corresponding author upon reasonable request.

## References

[1] Elendu C, Amaechi DC, Elendu TC, Ashna M, Ross-Comptis J, Ansong SO, et al. Heart failure and diabetes: understanding the bidirectional relationship. Medicine (Baltimore). 2023;102:E34906. 37713837 10.1097/MD.0000000000034906 PMC10508577

[2] Palazzuoli A, Iacoviello M. Diabetes leading to heart failure and heart failure leading to diabetes: epidemiological and clinical evidence. Heart Fail Rev. 2023;28:585. 35522391 10.1007/s10741-022-10238-6 PMC10140137

[3] Theofilis P, Oikonomou E, Tsioufis K, Tousoulis D. Diabetes mellitus and heart failure: epidemiology, pathophysiologic mechanisms, and the role of SGLT2 inhibitors. Life. 2023;13:497. 36836854 10.3390/life13020497 PMC9968235

[4] Sugandh F, Chandio M, Raveena F, Kumar L, Karishma F, Khuwaja S, et al. Advances in the management of diabetes mellitus: a focus on personalized medicine. Cureus. 2023;15:e43697. 37724233 10.7759/cureus.43697 PMC10505357

[5] Thomas MC. Type 2 diabetes and heart failure: challenges and solutions. Curr Cardiol Rev. 2016;12:249. 27280301 10.2174/1573403X12666160606120254 PMC5011193

[6] Htay T, Soe K, Lopez-Perez A, Doan AHA, Romagosa MA, Aung KK. Mortality and cardiovascular disease in type 1 and type 2 diabetes. Curr Cardiol Rep. 2019;21:1407. 10.1007/s11886-019-1133-9 31011838

[7] Bytyçi I, Bajraktari G. Mortality in heart failure patients. Anatol J Cardiol. 2015;15:63. 25550250 10.5152/akd.2014.5731 PMC5336901

[8] Colberg SR, Sigal RJ, Yardley JE, Riddell MC, Dunstan DW, Dempsey PC, et al. Physical activity/exercise and diabetes: a position statement of the American Diabetes Association. Diabetes Care. 2016;39:2065. 27926890 10.2337/dc16-1728 PMC6908414

[9] Flotyńska J, Szybiak W, Naskręt D, Zozulińska-Ziółkiewicz D, Grzelka-Woźniak A, Uruska A. Methods of Assessment of Physical Capacity in People with Diabetes Mellitus Type 1. Curr Diabetes Rev . 2024;20. 10.2174/1573399820666230608123917 37291777

[10] Herdy AH, Ritt LEF, Stein R, de Araújo CGS, Milani M, Meneghelo RS, et al. Cardiopulmonary exercise test: background, applicability and interpretation. Arq Bras Cardiol. 2016;107:467. 27982272 10.5935/abc.20160171 PMC5137392

[11] Heidenreich PA, Bozkurt B, Aguilar D, Allen LA, Byun JJ, Colvin MM, et al. 2022 AHA/ACC/HFSA guideline for the management of heart failure: a report of the American College of Cardiology/American Heart Association Joint Committee on Clinical Practice Guidelines. J Am Coll Cardiol. 2022;79:e263-421. 35379503 10.1016/j.jacc.2021.12.012

[12] Huang WM, Chang HC, Chen CN, Huang CJ, Yu WC, Cheng HM, et al. Symptom-limited exercise capacity is associated with long-term survival. Medicine (Baltimore). 2023;102:E34948. 37773832 10.1097/MD.0000000000034948 PMC10545336

[13] Zisman-Ilani Y, Fasing K, Weiner M, Rubin DJ. Exercise capacity is associated with hospital readmission among patients with diabetes. BMJ Open Diabetes Res Care. 2020;8:1771. 10.1136/bmjdrc-2020-001771 PMC7537144 33020136

[14] Nojima H, Yoneda M, Watanabe H, Yamane K, Kitahara Y, Sekikawa K, et al. Association between aerobic capacity and the improvement in glycemic control after the exercise training in type 2 diabetes. Diabetol Metab Syndr. 2017;9:63. 28828040 10.1186/s13098-017-0262-9 PMC5563031

[15] Myers J, Arena R, Cahalin LP, Labate V, Guazzi M. Cardiopulmonary exercise testing in heart failure. Curr Probl Cardiol. 2015;40:322–72. 26096801 10.1016/j.cpcardiol.2015.01.009

[16] Marinho RS, Jürgensen SP, Arcuri JF, Goulart CL, Dos Santos PB, Roscani MG, et al. Reliability and validity of six-minute step test in patients with heart failure. Brazilian J Med Biol Res. 2021;54:e10514. 10.1590/1414-431x2020e10514 PMC8289340 34287574

[17] Giannitsi S, Bougiakli M, Bechlioulis A, Kotsia A, Michalis LK, Naka KK. 6-minute walking test: a useful tool in the management of heart failure patients. Ther Adv Cardiovasc Dis. 2019;13:1–10. 10.1177/1753944719870084 PMC6710700 31441375

[18] Lee MC. Validity of the 6-minute walk test and step test for evaluation of cardio respiratory fitness in patients with type 2 diabetes mellitus. J Exerc Nutr Biochem. 2018;22:49. 10.20463/jenb.2018.0008 PMC5909078 29673246

[19] Poole DC, Richardson RS, Haykowsky MJ, Hirai DM, Musch TI. Exercise limitations in heart failure with reduced and preserved ejection fraction. J Appl Physiol. 2018;124:208. 29051336 10.1152/japplphysiol.00747.2017 PMC5866447

[20] Nesti L, Pugliese NR, Sciuto P, Natali A. Type 2 diabetes and reduced exercise tolerance: a review of the literature through an integrated physiology approach. Cardiovasc Diabetol. 2020;19:134. 32891175 10.1186/s12933-020-01109-1 PMC7487838

[21] Malta M, Cardoso LO, Bastos FI, Magnanini MMF, da Silva CMFP. STROBE initiative: guidelines on reporting observational studies. Rev Saude Publica. 2010;44:559–65. 20549022 10.1590/S0034-89102010000300021

[22] Nuttall FQ. Body mass index: obesity, BMI, and health: a critical review. Nutr Today. 2015;50:117. 27340299 10.1097/NT.0000000000000092 PMC4890841

[23] Carvalho VO, Guimarães GV, Carrara D, Bacal F, Bocchi EA. Validação da versão em português do Minnesota Living with Heart Failure Questionnaire. Arq Bras Cardiol. 2009;93:39–44. 19838469 10.1590/S0066-782X2009000700008

[24] Rohde LEP, Montera MW, Bocchi EA, Clausell NO, de Albuquerque DC, Rassi S, et al. Diretriz brasileira de insuficiência cardíaca crônica e aguda. Arq Bras Cardiol. 2018;111:436–539. 30379264 10.5935/abc.20180190

[25] De Castro Pereira CA, Sato T, Rodrigues SC. New reference values for forced spirometry in white adults in Brazil. J Bras Pneumol. 2007;33:397–406. 17982531 10.1590/s1806-37132007000400008

[26] Graham BL, Steenbruggen I, Barjaktarevic IZ, Cooper BG, Hall GL, Hallstrand TS, et al. Standardization of spirometry 2019 update. An Official American Thoracic Society and European Respiratory Society Technical Statement. Am J Respir Crit Care Med. 2019;200:e70. 31613151 10.1164/rccm.201908-1590ST PMC6794117

[27] Mitchell C, Rahko PS, Blauwet LA, Canaday B, Finstuen JA, Foster MC, et al. Guidelines for performing a comprehensive transthoracic echocardiographic examination in adults: recommendations from the American Society of Echocardiography. J Am Soc Echocardiogr. 2019;32:1–64. 30282592 10.1016/j.echo.2018.06.004

[28] McDonagh TA, Metra M, Adamo M, Gardner RS, Baumbach A, Böhm M, et al. 2021 ESC Guidelines for the diagnosis and treatment of acute and chronic heart failure: Developed by the Task Force for the diagnosis and treatment of acute and chronic heart failure of the European Society of Cardiology (ESC) With the special contribution of the Heart Failure Association (HFA) of the ESC. Rev Esp Cardiol (Engl Ed). 2022;75:523. 35636830 10.1016/j.rec.2022.05.005

[29] Arcuri JF, Borghi-Silva A, Labadessa IG, Sentanin AC, Candolo C, Di Lorenzo VAP. Validity and reliability of the 6-minute step test in healthy individuals: a cross-sectional study. Clin J Sport Med. 2016;26:69–75. 25706661 10.1097/JSM.0000000000000190

[30] Salles Albuquerque V, Dal Corso S, Pereira do Amaral D, Medina Dutrade Oliveira T, Fonseca Souza G, Naara Silva de Souza R, et al. Normative values and reference equation for the six-minute step test to evaluate functional exercise capacity: a multicenter study. J Bras Pneumol. 2022. 10.36416/1806-3756/e20210511. 10.36416/1806-3756/e20210511

[31] Anderson TW, Theodore W. An introduction to multivariate statistical analysis. 2003;721. Available from: https://www.wiley.com/en-us/An+Introduction+to+Multivariate+Statistical+Analysis%2C+3rd+Edition-p-9780471360919.

[32] Maldonado G, Greenland S. Simulation study of confounder-selection strategies. Am J Epidemiol. 1993;138:923–36. 10.1093/oxfordjournals.aje.a116813. 8256780 10.1093/oxfordjournals.aje.a116813

[33] Cohen J. Statistical power analysis for the behavioral sciences. Stat Power Anal Behav Sci. 2013. 10.4324/9780203771587. 10.4324/9780203771587

[34] JASP—Free and User-Friendly Statistical Software (version 0.18.2). Available from: https://jasp-stats.org/.

[35] Roh J, Hill JA, Singh A, Valero-Muñoz M, Sam F. Heart failure with preserved ejection fraction: heterogeneous syndrome, diverse preclinical models. Circ Res. 2022;130:1906–25. 35679364 10.1161/CIRCRESAHA.122.320257 PMC10035274

[36] Sullivan MJ, Hawthorne MH. Exercise intolerance in patients with chronic heart failure. Prog Cardiovasc Dis. 1995;38:1–22. 7631018 10.1016/S0033-0620(05)80011-8

[37] Tucker WJ, Haykowsky MJ, Seo Y, Stehling E, Forman DE. Impaired exercise tolerance in heart failure: role of skeletal muscle morphology and function. Curr Heart Fail Rep. 2018;15:323–31. 30178183 10.1007/s11897-018-0408-6 PMC6250583

[38] Zizola C, Schulze PC. Metabolic and structural impairment of skeletal muscle in heart failure. Heart Fail Rev. 2013;18:623–30. 10.1007/s10741-012-9353-8. 23065040 10.1007/s10741-012-9353-8 PMC3784612

[39] Bassi-Dibai D, Santos-de-Araújo AD, Dibai-Filho AV, de Azevedo LFS, Goulart CDL, Luz GCP, et al. Rehabilitation of individuals with diabetes mellitus: focus on diabetic myopathy. Front Endocrinol (Lausanne). 2022;13:869921. 35498435 10.3389/fendo.2022.869921 PMC9047902

[40] Bilak JM, Gulsin GS, McCann GP. Cardiovascular and systemic determinants of exercise capacity in people with type 2 diabetes mellitus. Ther Adv Endocrinol Metab. 2021;12. 10.1177/2042018820980235 PMC7844448 33552463

[41] Meyer K, Westbrook S, Schwaibold M, Hajric R, Lehmann M, Roskamm H. Cardiopulmonary determinants of functional capacity in patients with chronic heart failure compared with normals. Clin Cardiol. 1996;19:944–8. 8957598 10.1002/clc.4960191208

[42] Fuentes-Abolafio IJ, Stubbs B, Pérez-Belmonte LM, Bernal-López MR, Gómez-Huelgas R, Cuesta-Vargas AI. Physical functional performance and prognosis in patients with heart failure: a systematic review and meta-analysis. BMC Cardiovasc Disord. 2020;20:512. 33297975 10.1186/s12872-020-01725-5 PMC7724724

[43] Kuziemski K, Słomiński W, Jassem E. Impact of diabetes mellitus on functional exercise capacity and pulmonary functions in patients with diabetes and healthy persons. BMC Endocr Disord. 2019;19:2. 30606177 10.1186/s12902-018-0328-1 PMC6318966

[44] Ingle L, Reddy P, Clark AL, Cleland JGF. Diabetes lowers six-minute walk test performance in heart failure. J Am Coll Cardiol. 2006;47:1909–10. 16682322 10.1016/j.jacc.2006.02.005

[45] Berisha-Muharremi V, Henein MY, Dini FL, Haliti E, Bytyçi I, Ibrahimi P, et al. Diabetes is the strongest predictor of limited exercise capacity in chronic heart failure and preserved ejection fraction (HFpEF). Front Cardiovasc Med. 2022;9:883615. 35694665 10.3389/fcvm.2022.883615 PMC9178085

[46] Rozenbaum Z, Granot Y, Sadeh B, Havakuk O, Arnold JH, Shimiaie J, et al. Sex differences in heart failure patients assessed by combined echocardiographic and cardiopulmonary exercise testing. Front Cardiovasc Med. 2023;10:1098395. 36815019 10.3389/fcvm.2023.1098395 PMC9939638

[47] Herdy AH, Uhlendorf D. Reference values for cardiopulmonary exercise testing for sedentary and active men and women. Arq Bras Cardiol. 2011;96:54–9. 21109909 10.1590/S0066-782X2010005000155

[48] Dimopoulou I, Tsintzas OK, Daganou M, Cokkinos DV, Tzelepis GE. Contribution of lung function to exercise capacity in patients with chronic heart failure. Respiration. 1999;66:144–9. 10202318 10.1159/000029356

[49] Echouffo-Tcheugui JB, Xu H, DeVore AD, Schulte PJ, Butler J, Yancy CW, et al. Temporal trends and factors associated with diabetes mellitus among patients hospitalized with heart failure: findings from get with the guidelines-heart failure registry. Am Heart J. 2016;182:9–20. 27914505 10.1016/j.ahj.2016.07.025

[50] Zuin M, Rigatelli G, Battisti V, Costola G, Roncon L, Bilato C. Increased risk of acute myocardial infarction after COVID-19 recovery: a systematic review and meta-analysis. Int J Cardiol. 2023;372:138. 36535564 10.1016/j.ijcard.2022.12.032 PMC9755219

[51] Chagué F, Boulin M, Eicher JC, Bichat F, Saint Jalmes M, Cransac-Miet A, et al. Impact of lockdown on patients with congestive heart failure during the coronavirus disease 2019 pandemic. ESC Hear Fail. 2020;7:4420. 10.1002/ehf2.13016 PMC7537025 32997438

[52] Caraballo C, Desai NR, Mulder H, Alhanti B, Wilson FP, Fiuzat M, et al. Clinical implications of the New York Heart Association Classification. J Am Hear Assoc Cardiovasc Cerebrovasc Dis. 2019;8:e014240. 10.1161/JAHA.119.014240 PMC6912957 31771438

